# Immunotherapy in Myeloproliferative Diseases

**DOI:** 10.3390/cells9061559

**Published:** 2020-06-26

**Authors:** Lukas M. Braun, Robert Zeiser

**Affiliations:** 1 Department of Medicine I, Medical Center—University of Freiburg, Faculty of Medicine, University of Freiburg, 79106 Freiburg, Germany; lukas.braun@uniklinik-freiburg.de; 2 Faculty of Biology, University of Freiburg, 79104 Freiburg, Germany; 3 German Cancer Consortium (DKTK) Partner Site Freiburg, German Cancer Research Center (DKFZ), 69120 Heidelberg, Germany; 4 Comprehensive Cancer Center Freiburg (CCCF), University of Freiburg, 79106 Freiburg, Germany; 5 Centre for Biological Signalling Studies (BIOSS) and Centre for Integrative Biological Signalling Studies (CIBSS), University of Freiburg, 79104 Freiburg, Germany

**Keywords:** allo-HSCT, AML, CD123, IFNα, immune checkpoint, immunotherapy, inflammation, immune escape, JAK2, MDS, MDSCs, MPN, myeloproliferation, tumor vaccination

## Abstract

Myeloproliferative diseases, including myeloproliferative neoplasms (MPN) and myelodysplastic syndromes (MDS), are driven by genetic abnormalities and increased inflammatory signaling and are at high risk to transform into acute myeloid leukemia (AML). Myeloid-derived suppressor cells were reported to enhance leukemia immune escape by suppressing an effective anti-tumor immune response. MPNs are a potentially immunogenic disease as shown by their response to interferon-α treatment and allogeneic hematopoietic stem-cell transplantation (allo-HSCT). Novel immunotherapeutic approaches such as immune checkpoint inhibition, tumor vaccination, or cellular therapies using target-specific lymphocytes have so far not shown strong therapeutic efficacy. Potential reasons could be the pro-inflammatory and immunosuppressive microenvironment in the bone marrow of patients with MPN, driving tumor immune escape. In this review, we discuss the biology of MPNs with respect to the pro-inflammatory milieu in the bone marrow (BM) and potential immunotherapeutic approaches.

## 1. Introduction

The term “Myeloproliferative Disorders” describes non-physiological changes of the myeloid compartment in the hematopoietic system leading to an overproduction of mature and functional myeloid blood cells [1]. The WHO classification for hematopoietic tumors and Myeloproliferative Neoplasms (MPN) distinguishes different forms of the disease into chronic myeloid leukemia, chronic neutrophilic leukemia, chronic eosinophilic leukemia-not otherwise specified, primary myelofibrosis (PMF), polycythemia vera (PV), essential thrombocythemia (ET), and MPN unclassifiable (MPN-U) [2]. The three classical *BCR-ABL*-negative forms of MPN, which are the most frequent disorders among all myeloproliferative diseases, comprise PV, PMF, and ET. Somatic mutations cause a constitutive activation of physiologic signaling pathways in hematopoietic stem and progenitor cells, leading to a clonal expansion of myeloid progenitor cells and single or multilineage hyperplasia [3,4,5]. MPNs are characterized by an increased production of fully differentiated and completely functional blood cells of the myeloid lineage [1,5,6]. ET, PV, and PMF are clinically classified by an overproduction of functional platelets, increased numbers of red blood cells and high counts of white blood cells and bone marrow and spleen fibrosis, respectively [1,5].

The discovery of a novel *JAK2^V617F^* mutation, which is found in 50–90% of all classical MPNs and results in a substitution of valine to phenylalanine in the *JAK2* gene, significantly contributed to the discovery of the molecular pathogenesis of myeloproliferative neoplasms [5,7,8,9,10]. *JAK2* is the most-frequently mutated gene in MPN and its mutant form encodes a constitutively active kinase. The *JAK2^V617F^* mutation usually arises in a multipotent hematopoietic progenitor clone and can be found in all myeloid lineages, but also in B-, T- and NK-cells [5]. Another mutation of *JAK2* in exon 12 is found less frequently in MPNs and is mainly restricted to *JAK2^V617F^* negative PV [11]. Other more rarely seen genetic aberrations in MPN are mutations in the myeloproliferative leukemia virus (*MPL*; thrombopoietin receptor (*TPOR*)) gene resulting in a substitution of tryptophan at position W515 by leucine (*MPL^W515L^*) or lysine (*MPL^W515K^*) [12,13]. These mutations are not as common as *JAK2* mutations and are only found in 3–5% of all ET and PMF cases [14,15]. More recent discoveries found frameshift mutations in exon 9 in the calretikulin (*CALR*) gene in the majority of *JAK2-* and *MPL*-negative PMF and ET, causing a 5-bp insertion or a 52-bp deletion [5,16,17,18]. Genetic analyses could elucidate that the major MPN driver mutations, *JAK2^V617F^*, *CALR*, and *MPL^515^*, play a pivotal role in increasing the risk of leukemic transformations [19].

The transformation of Philadelphia-chromosome negative MPN to acute myeloid leukemia (AML) is one of the major complications of MPN [5,20]. Such a transformation of MPN to secondary AML is seen in about 1.5%, 4–7% and 11–20% for ET, PV, and PMF, respectively [3,4,19,21]. Standard therapeutic options are not effective in secondary AML transformed from MPN and patients suffering from a post-MPN AML only have a dismal prognosis with a median survival below 6 months [3,22,23]. The mechanisms driving leukemic transformation have not been well understood yet but it was seen that the risk of developing a secondary AML is increased by additional factors, e.g., age and chemotherapy treatment [3,4]. Also, genetic instability and acquired mutations are known as a risk factor for the development of a post-MPN AML [3]. Frequently reported genetic aberrations include mutations in epigenetic modifiers such as the Ten-Eleven Translocation 2 (*TET2*), isocitrate dehydrogenase 1 and 2 (*IDH1/2*), additional sex combs like transcriptional regulator 1 (*ASXL1*) and enhancer of zeste 2 polycomb repressive complex 2 subunit (*EZH2*), mutations in spliceosome regulators such as serine and arginine rich splicing factor 2 (*SRSF2*), splicing factor 3B subunit 1 (*SF3B1*) and U2 small nuclear RNA auxiliary factor 1 (*U2AF1*) and genetic aberrations in the DNA damage control system, including *TP53* [24,25,26,27,28,29,30,31,32,33,34]. Additional mutations were found in the protein tyrosine phosphatase non-receptor type 11 (*PTPN11*), *MYC* and the SET binding protein 1 (*SETBP1*) [3].

## 2. Myeloproliferative Diseases are Driven by Inflammation

Many publications described that myeloproliferative diseases are driven by activation of inflammatory pathways leading to increased levels of cytokines and accumulation of reactive oxygen species (ROS) [35]. The latter were reported to play an important role in JAK2-mutant MPN progression as ROS was seen to accumulate in the hematopoietic stem-cell compartment of *JAK2^V617F^* knock-in mice and was found increased in patients with *JAK2^V617F^* mutant MPN [36]. According to these findings, transcriptional profiling of peripheral blood samples from MPN patients revealed a significant deregulation of anti-oxidative stress genes, e.g., *Nrf2*, in MPN patient samples compared to controls [37]. Regarding the overproduction of ROS in the hematopoietic stem-cell compartment of *JAK2^V617F^* knock-in mice, the application of the anti-oxidant N-acetylcysteine (NAC) could restore the normal phenotype in these mice, normalize peripheral blood parameters, decrease splenomegaly, reduce the number of *JAK2*-mutant hematopoietic stem and progenitor cells in the spleen and bone marrow and reduce DNA double-strand breaks being increased in *JAK2^V617F^* mutant MPN. The authors claimed that the massive production of ROS in *JAK2^V617F^*-mutant MPN causes DNA damage, thereby driving disease progression and the development of MPN could be slowed down by specifically targeting ROS [36].

Regarding increased pro-inflammatory signaling, one publication reported the oncogenic *KRAS^G12D^* mutation as a driver for elevated myeloproliferation and chronic myelomonocytic leukemia (CMML) through activation of the NLRP3 inflammasome and caspase-1-mediated cleavage of pro-inflammatory cytokines [38,39]. Underlining the notable role of inflammasome activation for driving myeloproliferation, a genetic deficiency of *NLRP3* could ameliorate *KRAS^G12D^* driven cytopenia in mice [39]. Moreover, additional studies could highlight that *MPL^W15L^* mutant mice showed high serum levels of pro-inflammatory cytokines including Interleukin-6 (IL-6), tumor necrosis factor (TNF) α, IL-10, CXCL9 and CXCL10 [40,41]. Comparable, the oncogenic *JAK2^V617F^* mutation caused high levels of IL-6 and TNFα in the serum of mice being transplanted with a *JAK2^V617F^* overexpressing cell line or carrying the mutation in the bone marrow [40,42]. Besides the major MPN mutations, also other genetic aberrations can increase the release of pro-inflammatory cytokines, thereby potentially driving the progress of the disease. One study highlighted the role of pro-inflammatory signaling pathways in driving the expansion of pre-leukemic hematopoietic stem and progenitor cells (HSPCs). It was shown that *TET2*-deficiency caused an increased IL-6 production which in turn activated the Shp2/STAT3 signaling axis leading to higher anti-apoptotic and pro-survival protein levels in HSPCs [43]. Moreover, additional studies described that *TET2*-deficiency drives the production of pro-inflammatory cytokines, including interleukin 1β (IL-1β) and interleukin 18 (IL-18) [44,45,46]. Besides *TET2*, mutations in other epigenetic modifiers, e.g., *DNMT3A* and *ASXL1*, which are frequently mutated in pre-leukemic hematopoiesis, were reported to drive an increased inflammatory signaling by secreting IL-6 and TNFα [46,47,48]. Further studies could prove a pivotal role of TNFα and interferon α (IFNα) secretion and the activation of pro-inflammatory signaling pathways for driving myelofibrosis in *EZH2*/*JAK2^V617F^*-mutant myeloid progenitor cells or increased TNFα signaling in *DNMT3A/JAK2^V617F^*-mutant myeloid stem and progenitor cells [49,50]. Activated TNFα pathways were also confirmed by RNA-seq analysis in patients suffering from *EZH2*- or *DNMT3A*-deficient myelofibrosis compared to non-mutant controls [49]. Hemmati et al. did propose the importance of an activated inflammatory signaling for driving pre-leukemic myeloproliferative diseases and leukemic transformations [46].

Most of the up-regulated inflammatory signaling molecules are reported to stimulate the JAK/STAT signaling axes, thereby increasing the viability, proliferation and survival of many different cell types, including malignant cancer cell clones [51,52,53]. However, pro-inflammatory signaling is not only up-regulated in pre-malignant and malignant MPN cell clones but also in niche cells, neutrophils, monocytes, and endothelial cells in the tumor microenvironment, thereby significantly contributing to tumor immune escape [40,42,54]. The stimulation of JAK2 signaling pathways again promotes the production of more inflammatory cytokines by activating nuclear factor-κ-B (NFκB) signaling [55,56].

Within the three major types of MPN, genetic abnormalities drive neutrophil gene expression causing an activation of inflammatory signaling pathways with elevated secretion of IL-6, IL-1β, interleukin-8 (IL-8), interleukin-11 (IL-11), interleukin-17 (IL-17), TNFα, transforming growth factor β (TGFβ) or granulocyte-macrophage colony-stimulating factor (GM-CSF) [4,43,57,58,59,60,61,62]. Therapeutic blockade of constitutively activated JAK1/2 signaling by Ruxolitinib treatment could decrease the inflammatory signature in the serum of MPN patients, showing that the underlying mutations cause and activation of pro-inflammatory signaling [63,64].

**Figure 1:**
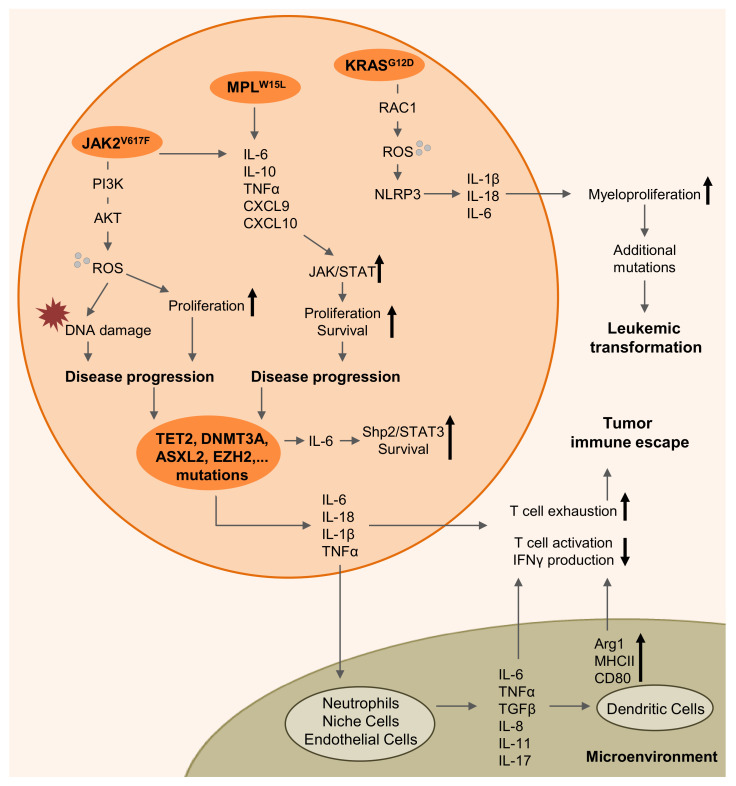
Pro-inflammatory signaling processes driving myeloproliferation and leukemia immune escape in myeloid malignancies. Oncogenic mutations stimulate increased production of ROS and pro-inflammatory cytokines and interleukins. ROS causes DNA damage and favors proliferation of the mutant clone, thereby driving disease progression. Cytokines drive disease progression through elevated Shp2/STAT3 and JAK/STAT signaling. NLRP3-Inflammsome activation results in enhanced myeloproliferation, driving leukemic transformation of myeloproliferative diseases. Increased cytokine signaling in the tumor microenvironment contributes to T-cell exhaustion, reduced T-cell activation, and leukemia immune escape.

In 2018, Kaplanov et al. clearly demonstrated in a breast cancer model that increased levels of IL-1β in the tumor microenvironment are responsible for immunosuppression [65]. Although they did observe tumor progression and spontaneous metastasis in WT mice orthotopically transplanted with 4T1 breast cancer cells, tumors began to grow but regressed and did not form metastasis in IL-1β-deficient recipients. The mechanism is based on a reduced monocyte infiltration and a lower differentiation in IL-1β-deficient mice. Moreover, macrophages secrete immunosuppressive IL-10 in WT mice, whereas IL-12 secreted by dendritic cells in mice lacking IL-1β expression stimulates anti-tumor immunity. Following IL-1β loss or blockade, CD8^+^ T-cells are activated, express higher levels of the effector molecules IFNγ, TNFα and granzyme B and synergize with immune checkpoint blockade [65]. Also recent data indicate that oncogenic *KRAS* leads to NLRP3 activation and IL-1β production which promotes myeloproliferation [39]. Besides IL-1β signaling, increased levels of IL-6 are known to be a poor prognostic factor for a variety of tumors [66]. For a long time, IL-6 was thought to mediate its negative effects through the JAK/STAT, PI3K/Akt and Ras/MAPK signaling pathways, but it is also known that IL-6 has manifold immunomodulatory effects [66,67,68,69]. Increased levels of IL-6 were found responsible for impaired Th1 differentiation and responses and for causing an inadequate CD4^+^ helper T-cell activity for CD8^+^ T-cells, resulting in limited tumor elimination [70,71,72]. Regarding the myeloid compartment, increased IL-6 signaling could aid to enhance the expression of immunosuppressive arginase-1 or to diminish major histocompatibility complex II (MHCII) and CD80 expression in dendritic cells (DCs), thereby supporting tumor immune escape mechanisms [73,74,75]. Both cytokines are an example on how increased inflammatory signaling can not only stimulate immune responses, but also dampen an effective anti-tumor immune response. Figure 1 summarizes the inflammatory signaling cascades driving myeloproliferation, disease progression, leukemic transformation, and tumor immune escape.

## 3. Allogeneic Hematopoietic Stem-Cell Transplantation

For many different myeloid malignancies, including MPN, MDS, and AML, allogeneic hematopoietic stem-cell transplantation (allo-HSCT) is the only potentially curative therapy. Since many myeloid malignancies are clonal disorders, a removal of the diseased clone by a conditioning regimen can eliminate the malignant stimulus and cure fibrosis, pro-inflammatory signaling and disease progression which is driven by mutant cells [76]. Most importantly, for MPN patients being at high risk of progressing and transforming into AML, allo-HSCT is the only curative modality if carried out before transformation [77]. The 5-year survival rate after allo-HSCT ranges from 30% to 70% and the regimen was reported to have the potential to achieve a resolution of bone marrow fibrosis [77,78]. A graft-versus-myelofibrosis effect could be increased by donor lymphocyte infusion (DLI) for patients relapsing after allo-HSCT and the bone marrow fibrosis was reverted within 12 months after transplantation [76,79]. Although allo-HSCT can offer long-term relapse-free survival, it is associated with high mortality and morbidity in patients with myelofibrosis (MF) and the therapeutic approach is only available for about 30% of all patients [80,81,82]. For patients diagnosed with low-risk disease, allo-HSCT is not necessarily indicated as the best option and should rather be reserved for disease progression [83]. Patients undergoing allo-HSCT were more prone to engraftment failure if having severe bone marrow fibrosis, whereas the engraftment was good in patients with only mild or moderate marrow fibrosis. The rate of failure was 33% versus 6% in these cohorts, respectively [77,84]. Besides MPN, allo-HSCT is currently also the only curative treatment option for patients suffering from MDS with long-term survival rates ranging from 25% to 70% [85,86,87,88]. Comparable to allo-HSCT for the treatment of MPN and MF patients, the regimen does also come along with the risk of treatment-related mortality, severe toxicities, and fatal complications in MDS. These strong side effects must be carefully weighed against the potential benefit of transplantation [85]. It was reviewed that patients matching the appropriate performance status and donor criteria should directly undergo allo-HSCT rather than undergoing conventional treatment [77]. Moreover, postponing allogeneic transplantations until patients are in a more advanced disease state did result in a worse outcome and a treatment failure [77,89,90]. The selection of hematopoietic stem-cell donors is crucial for engraftment and the treatment success. Since only 25–30% of all patients have HLA-matched sibling donors, other stem-cell sources such as umbilical cord blood or HLA-haploidentical donors, or even HLA-mismatched donors have to be used. However, the use of alternative donor sources other than HLA-matched siblings is often accompanied by severe graft-versus-host disease (GvHD) and it needs to be further evaluated in terms of effectiveness and success rates to treat MPN patients [77]. Post-transplant relapse, graft failure and GvHD remain the major cause of treatment failure [91]. It is crucial to enhance the graft-versus-leukemia (GvL) effect while minimizing the risk of GvHD. The risk of development of severe GvHD mostly depends on HLA-matching, the stem-cell source, the conditioning regimen and GvHD prophylaxis following transplantation [85]. Patients relapsing after allo-HSCT have only very poor survival. About 32% of all AML patients receiving reduced intensity conditioning relapsed after allo-HSCT and the 2-year survival rate after relapse was only at 14% [92]. Additional DLI was reported to enhance patient survival, but did also lead to more severe GvHD [91]. Novel strategies to reduce severe GvHD while preserving the GvL effect include the application of hypomethylating agents (HMA), e.g., Azacitidine, as transplantation prophylaxis or together with DLI which was reported to reduce GvHD through higher regulatory T-cells (T_reg_) numbers. Besides an increase of regulatory T-cells, HMA treatment was reported to enhance cytotoxic T-cell reactivity against different tumor antigens, thereby increasing the anti-tumor immune response [93,94].

Besides allo-HSCT, JAK2 inhibition using Ruxolitinib is currently the only promising therapeutic option for MF patients and is also reviewed in the following chapters. Clinical studies could show that JAK2 inhibition is a promising strategy to reduce the spleen size and increase the survival of MPN patients [95]. It was therefore hypothesized that Ruxolitinib treatment before allo-HSCT could be a novel strategy to significantly improve patient survival after transplantation [77]. Jaekel et al. showed that JAK1/2 inhibition prior to HSCT significantly decreased MF-related symptoms and splenomegaly in most of all analyzed patients. Moreover, stem-cell engraftment was seen in 93% of all patients, whereas severe GvHD was only reported for 14% of all patients if treated with Ruxolitinib. It was concluded that JAK1/2 inhibition could improve the engraftment and outcome after allo-HSCT in MF patients through reduction of pro-inflammatory signaling and reduction of splenomegaly [96]. These findings clearly indicate that combinatorial treatments are promising and novel strategies to overcome the unmet need for successful therapies for MPN patients.

In summary, many different factors determine the outcome of patients suffering from hematopoietic malignancies after allo-HSCT. These include the age, disease state, symptom burden, genetic aberrations, and pre-treatments of the recipient, but also the timing of transplantation, the donor source, HLA-matching, and the conditioning regimen. Moreover, post-transplant and relapse handling is crucial for patient outcome [97]. Although the outcome of patients after allo-HSCT was continuously improved during recent decades, disease recurrence and severe GvHD remain the major problems which are tried to be controlled by additional therapeutic regimens [85]. Besides allo-HSCT, many more therapeutic concepts were arising during recent years trying to overcome the unmet need for novel and effective therapies for the treatment of patients suffering from myeloid malignancies.

## 4. MDSCs Mediate Leukemia Immune Escape

Myeloid-derived suppressor cells (MDSCs) were previously described as being able to suppress a strong anti-leukemia immune response in patients, thereby supporting tumor immune escape [98]. MDSCs are a heterogeneous population of myeloid cells which can suppress the activity and the anti-leukemia immune response of T-cells through a variety of mechanisms either via direct cell–cell contact or by the release of soluble factors [98,99,100]. The best understood mechanisms include increased production of ROS, an increased expression of arginase-1 and inducible nitric oxide synthase (iNOS), the secretion of peroxynitrite and the promotion of de novo development of regulatory T-cells [99,100,101,102,103]. L-arginine significantly contributes to the immunosuppressive effect of MDSCs, as it serves as a substrate for both arginase-1 and iNOS [100]. Nitric oxide (NO) was reported to limit T-cell functions through reduced MHCII surface expression and increased T-cell death [104,105]. A high expression of arginase-1 results in the depletion of L-arginine in the tumor microenvironment, thereby inhibiting the proliferation of NK-cells and the proliferation and IFNγ production of cytotoxic T-cells [106,107,108,109]. Steggerga et al. highlighted the pivotal role of arginase-1 in suppressing an effective anti-tumor immune response. Blockade of arginase-1 using a small molecule inhibitor did reverse the myeloid cell-mediated suppression of T-cell activity and proliferation and did reduce the growth of melanoma, lung cancer and breast tumors in vivo due to increased NK and T-cell infiltration into the tumor microenvironment [109].

Many studies could link increased numbers of MDSCs with the development of myeloid disorders, including MDS and MPN [110,111]. Chen et al. confirmed that MDSCs are significantly increased in the bone marrow of MDS patients compared to age- and gender-matched healthy control or non-MDS cancer specimens. Moreover, it was highlighted that increased CD33 expression on MDS-MDSCs contributes to the suppression of normal myeloid cell development. Knockdown of CD33 on MDSCs reduced the secretion of TGF-β, IL-10, and arginase-1 activity, thereby decreasing their immunosuppressive activity [110]. Besides MDS, MDSCs were also found to be significantly increased in MPN patient peripheral blood compared to healthy donor controls. There were no differences found between the different forms of MPN. Comparable with previously published results, MDSCs freshly isolated from MPN patients did inhibit the proliferation of CD3^+^ T-cells and showed an increased expression of arginase-1 [98].

One strategy to overcome MDSC-induced immune suppression is the application of the demethylating agent Decitabine which was shown to significantly deplete MDSCs in vivo through induction of apoptosis. Treatment of Decitabine did preferentially induce cell death in MDSCs, whereas healthy immune cells were only affected at higher doses. An in vitro mixed lymphocyte reaction did confirm that the T-cell proliferation is enhanced upon application of Decitabine and the findings were confirmed in an in vivo leukemia model [112]. A phase I/II study is aiming to evaluate the potential of a Ruxolitinib and Decitabine combination treatment in relapsed or refractory and post-MPN AML patients (ClinicalTrials.gov Identifier: NCT02257138). Since both compounds were reported to have single-agent activity and it was shown that the combination could suppress the colony formation of post-MPN AML cells in vitro, the investigators hypothesize that combining Decitabine and Ruxolitinib could be beneficial for post-MPN AML treatment in patients who represent a population with an unmet medical need [3,113]. An additional epigenetic targeting approach combining Ruxolitinib with bromodomain and extraterminal (BET) inhibitors was reported to be effective in pre-clinical studies using post-MPN AML cell lines and primary patient cells [114]. However, at time of review writing, no results of these studies were published yet.

## 5. Interferon Alpha

Myeloproliferative disorders (MPDs) are potentially immunogenic neoplasms, as demonstrated by their susceptibility to recombinant interferon-α-2a (rIFNα-2a) [115,116,117]. A first study could describe the application of rIFNα-2a to be an effective treatment strategy for controlling thrombocytosis in MPDs by decreasing platelet counts in patients within two to ten weeks after treatment [115]. More studies followed strengthening the outstanding role of long-term IFNα application in causing major molecular remission in *JAK2^V617F^*-mutant MPN patients [118]. Clinical studies found that pegylated IFNα is safe and well tolerated to use in PV and MF patients (ClinicalTrials.gov Identifier: NCT02910258, NCT00241241) [119,120]. All described clinical trials for IFNα-2a therapy in MPN are summarized in Table 1. The application of pegylated IFNα did cause a significant decrease of JAK2 allelic burden in *JAK2^V617F^*-mutant PV patients and after one year of treatment, all patients showed a hematologic and molecular response, including more than 90% of them being in complete remission with only mild adverse events (AEs) [119,120,121]. The mechanisms by which IFNα acts to control the disease are manifold and includes anti-proliferative, pro-apoptotic and immunomodulatory effects [122,123]. IFNα-2a binds to IFNα receptor chains and activates intracellular JAK signaling pathways, leading to a translocation of transcription factors into the nucleus [123]. Moreover, IFN application was shown to have an inhibitory effect on telomerase activity in leukemia cells [124]. Different studies of Riley et al. highlighted the immunomodulatory potential of IFNα by reducing the number of tumor cells through an increase of CD56^bright^ NK-cells and a reduction of CD56^dim^ NK-cells in the peripheral blood in patients with *JAK2^V617F^*-mutant MPN, therewith enhancing the anti-tumor immune response against *JAK2*-mutant MPN clones [118,125]. However, the treatment with IFNα also has limitations due to an inflammation-based degradation of IFNAR1, one of the type I IFN receptor chains necessary for IFNα binding. In a study on melanoma cells, the inflammatory cytokines interleukin 1α (IL-1α) and TNFα, secreted by melanoma cells, but no other cytokines, were reported to stimulate the phosphorylation, and subsequent ubiquitination and degradation of IFNAR1 [126]. Additionally, the induction of oxidative stress did block JAK/STAT signaling pathways in hepatitis patients, thereby reducing the antiviral gene expression which is normally induced upon INFα application [127]. As explained, MPDs are accompanied by an increased production and high serum levels of pro-inflammatory cytokines produced by the malignant MPN clone itself and by the tumor microenvironment [128]. One could now argue that interferon treatment is only a limited option in patients with late stage MPN, mostly in MF patients, due to an enhanced production of inflammatory cytokines in the bone marrow stroma cells [129]. A reduction of inflammatory cytokines might be a promising strategy to overcome the inflammation-induced limitation of IFNα treatment and a combination treatment with IFNα together with Ruxolitinib [129]. Ruxolitinib is a JAK1/2 inhibitor which has potent anti-inflammatory activity and is safe to combine with IFNα treatment, proven by clinical testing [130,131,132]. The RUXOPeg clinical study is currently recruiting patients with PMF, post-PV MF or post-ET MF to evaluate the combination of Ruxolitinib with pegylated IFN-alpha-2a (PEG-IFNα-2a) for its treatment safety and efficacy and the molecular response. However, the study is still recruiting and there were no results published yet (ClinicalTrials.gov Identifier: NCT02742324). Another clinical trial aimed to elucidate the potential of pegylated IFNα-2a compared to Hydroxyurea (HU) in PV and ET patients (ClinicalTrials.gov Identifier: NCT01259856) [133]. They recruited patients previously treated with HU and found that pegylated IFNα-2a achieves overall response rates of 69% and 60% in ET and PV patients, respectively. Moreover, they associated the presence of *CALR* mutations with better complete remission rates in ET patients upon treatment with pegylated IFNα-2a. Since the observed high rate of side effects was manageable in most patients, the investigators claimed that this therapy could be a promising therapy for ET and PV patients being refractory or resistant to HU treatment [133].

## 6. JAK2 Inhibition

Since the majority of all MPN is driven by *JAK2^V617F^* mutations, it was standing to reason to identify potent JAK2 inhibitors, of which Ruxolitinib is currently the only clinically approved compound [134]. Ruxolitinib is a tyrosine kinase inhibitor inhibiting both JAK1 and JAK2, and was approved for the treatment of intermediate and high-risk MF and as second-line therapy for PV refractory to HU treatment. Application of Ruxolitinib did proof its efficacy in reducing spleen volume, increasing overall survival (OS) and reducing symptom burden in general. A summary of the COMFORT-I and COMFORT-II clinical trial results highlighted that Ruxolitinib treatment significantly reduced splenomegaly, alleviated symptoms, reduced the death rate, and improved OS of MF patients. However, the treatment efficacy is variable throughout the different MPN phenotypes. For PV patients being resistant to HU treatment, the phase III trials RESPONSE I and II confirmed better disease control, splenomegaly reduction and a reduction of symptoms upon Ruxolitinib treatment [51]. Prolonged treatment with Ruxolitinib did reduce the risk of worsening fibrosis in some patients, did improve symptoms and bone marrow morphology, but curation from the underlying disease was rare [135,136,137,138]. The effects on how JAK2 inhibition contributes to a better disease control are manifold, but the best understood are the strong proliferation inhibition and the anti-inflammatory properties. Ruxolitinib treatment was proven to have a good efficacy to reduced inflammatory signaling in patients suffering from GvHD [130] and it did also reduce the concentration of pro-inflammatory cytokines in the serum of MPN patients through blockade of JAK/STAT signaling, thereby improving the clinical response of these patients [139]. One clinical trial did also link another JAK2 inhibitor with decreased inflammatory cytokines, reduced splenomegaly and the exertion of immunomodulatory effects (ClinicalTrials.gov Identifier: NCT01437787) [140]. Since Ruxolitinib does reduce the production of pro-inflammatory cytokines important for dendritic cell differentiation needed to activate T-cells, there were reports highlighting that Ruxolitinib causes an immunosuppressive effect and an increased risk of infections. Also, the proliferation of cytotoxic T-cells was reduced upon Ruxolitinib application [141,142]. The reduction of T-cell and NK cell numbers might be one reason for an increase of different infections, e.g., reactivation of toxoplasma retinitis, tuberculosis, and hepatitis B, seen in Ruxolitinib treated patients [143,144,145]. Nevertheless, the overall incidence of infections and infectious AEs was low and acceptable in MPN patients in most clinical trials and the effects of JAK2 inhibition in reducing MPN symptoms in patients were superior to possible AEs [146,147,148].

Studies were ongoing to evaluate if Ruxolitinib could be combined with any other known MPN treatment strategy to improve the treatment outcome. One study combined the previously described compound IFN-α2 with Ruxolitinib to overcome the inflammation-mediated toxicity which limits the use of IFN-α2 in 10–30% of all patients. The underlying phase II study did prove in a cohort with 50 MPN patients, of which 47 were resistant or intolerant to IFN-α2 monotherapy, that a combination therapy with pegylated IFN-α2 and Ruxolitinib could indeed reduce the *JAK^V617F^* allelic burden and cause complete or partial remission (9% in PV, 39% in MF) or sustained complete hematologic response (44% in PV, 58% in MF). AEs were seen in up to 50% of the patients, but were manageable by dose reduction. Treatment had to be discontinued in 20%. The investigators concluded that a combination therapy could be beneficial for patients with low-risk MF and some PV patients. However, more clinical studies are needed to support these findings [132].

## 7. Targeting CD123

Interleukin-3 is part of a discrete family of cytokines regulating growth, differentiation, and migration of hematopoietic cells and signals through heterodimeric cell surface receptors, dimerized from CD123 (alpha chain of the interleukin-3 receptor, IL-3R-α) and CD131 (common beta chain, βc) [149,150,151]. However, the overexpression of this cytokine family or its receptors can initiate excessive signaling resulting in pathological events like inflammatory diseases or MPN and myeloid leukemia [151]. IL-3 signaling is initiated by its binding to CD123, followed by the recruitment of CD131 and the assembly of the receptor complex, triggering downstream signaling through JAK2 [149]. The expression of CD123 in the hematopoietic compartment, particularly on the surface of stem and progenitor cells from healthy individuals and AML patients, was extensively studied [152]. CD123 expression was found to be highly expressed on CD34^+^ stem and progenitor cells and blasts derived from AML patients [150,153]. Although CD123 is expressed on CD34^+^CD38^−^ AML cells, the normal CD34^+^CD38^−^ bone marrow counterpart did not express CD123 [154]. Since these CD34^+^CD38^−^CD123^+^ cells were able to engraft and recapitulate the leukemic disease in immunodeficient mice, these cells are seen as leukemic stem cells (LSCs) [154]. The overexpression of CD123 on AML cells is associated with a negative prognosis, increased cell numbers, higher cell-cycle activity, reduced apoptosis signaling and constitutive phosphorylation of STAT5 [155]. Based on these findings and the fact that CD123 is only expressed by malignant cell clones, CD123 was found as a promising suitable target molecule to attack AML cells without affecting normal hematopoietic cells.

One strategy to target leukemic stem cells via compounds binding to CD123, the immunotoxin Tagraxofusp (SL-401) was invented. SL-401 is a recombinant protein of IL-3 fused to the catalytic and translocation domains of diphtheria toxin and was FDA approved in December 2018 for the application in pediatric and adult blastic plasmacytoid dendritic cell neoplasm (BPDCN) [156]. The compound binds with high affinity to the IL-3 receptor (CD123) on malignant LSCs and is subsequently internalized via receptor-mediated endocytosis and transported to the endosome. The fragment containing the catalytic domain of diphtheria toxin is cleaved and translocated into the cytosol to inactivate elongation factor 2 (EF2) which is essential for protein synthesis. The protein synthesis is inhibited and the LSCs are thereby driven into apoptosis [156,157]. The combination of IL-3 and diphtheria toxin has shown robust activity in hematologic malignancies, e.g., BPDCN, in pre-clinical animal models and clinical trials [158,159]. A phase II clinical trial is currently ongoing to evaluate the potency of a targeted therapy to CD123 using the compound Tagraxofusp in patients with CMML or MF (ClinicalTrials.gov Identifier: NCT02268253). Stage 1 of the study is closed and evaluated the highest tolerated dose. Patients with CMML and MF are currently recruited in stage 2 of the study and treated with the dose of Tagraxofusp found in stage 1 (12 mcg/kg) to evaluate the response rate. In this study, 27 patients with MPN were treated, whereas 14 of these patients had previously received ≥3 lines of therapy. 53% of patients with evaluable baseline splenomegaly were reported with spleen size reduction, including 4 patients with reductions >45%. In summary, the symptom response rate was 45%. The most common treatment-related adverse events (TRAEs) included thrombocytopenia, anemia, headache, and hypoalbuminemia, whereas thrombocytopenia and anemia were the most common ≥ grade 3 TRAEs [153]. All trials about anti-CD123 therapies are summarized in Table 2. Two additional clinical trials about the use of Tagraxofusp in hematologic malignancies were launched in early 2020. One phase II trial is aiming to study the effects of the immunotoxin Tagraxofusp in treating patients with BPDCN after auto- or allo-HSCT (ClinicalTrials.gov Identifier: NCT04317781). The primary objective is to evaluate the safety of the treatment in this disease and secondary aims are to estimate progression-free survival and overall survival in patients with BPDCN receiving a maintenance therapy with Tagraxofusp after allo- or auto-HSCT. The second new phase II clinical trial is aiming to treat patients with relapsed/refractory CD123^+^ AML or with Blastic Plasmacytoid Dendritic Cell Neoplasm Immunophenotype-like (BPDCN-IPh-like) AML with Tagraxofusp (ClinicalTrials.gov Identifier: NCT04342962). At the time of this review writing, both of the above-mentioned clinical trials were recruiting patients and did not publish any results yet.

Besides the immunotoxin Tagraxofusp, CD123 can also be specifically targeted with therapeutic antibodies. In a pre-clinical murine xenograft model, Jin et al. could highlight the curative potential of a monoclonal antibody directed against CD123 to eliminate AML leukemia stem cells [160]. They reported that application of this antibody impairs the homing of leukemia stem cells to the bone marrow and their engraftment and that antibody treatment activates the innate immune system of the xenograft recipients. Mice treated with anti-CD123 showed reduced AML burden and reduced secondary transplantation capacity [160]. Nievergall et al. described in 2014 the potential of a humanized monoclonal antibody against CD123 (CSL362) for the treatment of chronic myeloid leukemia (CML) through depletion of CM progenitor and stem cells [161]. Although tyrosine kinase inhibitors (TKI) are effective in CML therapy, the remaining LSCs are hard to treat and may survive the treatment. Since CD123 was also found to be highly expressed on CD34^+^CD38^−^ LSCs in blast crisis and chronic phase CML patients compared to healthy donors, these cells were targetable in CML patients. Indeed, infusion of anti-CD123 antibody in mice did diminish leukemia engraftment due to a selective antibody-dependent cell-mediated cytotoxicity (ADCC)-facilitated lysis of LSCs. The ADCC response was mainly promoted by allogeneic and autologous NK-cells. The authors did also nicely highlight the synergistic effects of TKIs together with anti-CD123 monoclonal antibodies to reduce CML progenitor cells without effecting normal HSPCs [161]. A second publication did underline the efficacy of the anti-CD123 monoclonal antibody CSL362 to reduce AML growth and to deplete LSCs and AML blasts in AML xenograft mouse models through a potent induction of NK cell-mediated ADCC. The therapy was also effective against plasmacytoid dendritic cells (pDCs) and basophils in cynomolgus monkeys [162]. The Immunologic effects behind CSL362-mediated ADCC against AML cells and LSCs are based on NK-cells. Busfield et al. described that CSL362 is engineered to bind with increased affinity to CD16 and they clearly demonstrated that NK-cells are the effector cells using PBMCs, purified NK-cells or NK cell depleted PBMCs as effector compartment in co-culture with AML cells [162]. Numerous clinical studies are currently ongoing to evaluate the potential of anti-CD123 antibodies for the treatment of myeloid malignancies. A first-in-human phase I clinical trial was launched in 2012 to investigate the safety, pharmacodynamics and -kinetics, as well as the immunogenicity of repeated doses of CSL362 in patients with CD123^+^ AML currently in complete remission or complete remission with incomplete platelet recovery at high risk of early relapse (ClinicalTrials.gov Identifier: NCT01632852). TRAEs against CSL362 in ≥10% of the patients were infusion reactions, hypertension, hypotension, and increase in C-reactive protein. Basophils and pDCs were depleted in patients within the first six hours after treatment application and depletion sustained for at least 15 days post-treatment and pro-inflammatory cytokines significantly increased after the first applied dose of CSL362. The authors did conclude that CSL362 is well tolerated and safe to use in AML patients with complete remission and complete remission with incomplete platelet recovery at high risk of early relapse [163]. Eleven patients were recruited into the trial with minimal residual disease (MRD^+^) status and CSL362 application could convert MRD^+^ to MRD^−^ status in 4 of these 11 patients at 24 weeks follow-up. These findings suggested that CSL362 did eradicate residual LSCs. Nevertheless, it is thought that treatment efficacy of CSL362 immunotherapy against AML is depending on many different factors including molecular mutations, cytogenetics, the patient’s immune system, target expression on LSCs and number and activity of NK-cells [164]. Another humanized monoclonal anti-CD123 antibody, JNJ-56022473 (Talacotuzumab), was derived from CSL362 and tested in vitro in AML cell lines and was found to potently mediate cytotoxic activity [165]. One phase II clinical trial evaluated an immunotherapy-based approach with JNJ-56022473 as a single agent in MDS and AML patients being refractory to hypomethylating agents (HMA). However, the study was terminated due to recommendations by FDA. A second trial was conducted to evaluate the efficacy and safety of Decitabine together with Talacotuzumab in AML patients who cannot undergo intensive chemotherapy treatment. However, the combination treatment did not show any superior effect over Decitabine alone (ClinicalTrials.gov Identifier: NCT02472145). SGN-CD123A is an antibody–drug conjugate (ADC) using a linker molecule together with a humanized anti-CD123 antibody. Upon application in vitro, SGN-CD123A induced DNA damage, cell-cycle alterations and apoptosis in CD123^+^ AML cell lines and primary samples from AML patients. Moreover, various in vivo studies did underline its efficacy against AML in xenograft models and its ability to reduce AML cell growth [166]. The safety profile, maximum tolerated dose and efficacy of this antibody was evaluated in a clinical trial in patients with refractory or relapsed AML. However, the study was terminated, and no results were published yet (ClinicalTrials.gov Identifier: NCT02848248). According to in vitro and pre-clinical in vivo data, one fully human anti-CD123 monoclonal antibody, KHK2823, was thought to be a promising anti-CD123 therapeutic therapy against AML, MDS and B-ALL [167]. However, a clinical trial investigating KHK2813 in patients with relapsed or refractory AML and MDS was conducted but recently terminated due to failed treatment response (ClinicalTrials.gov Identifier: NCT02181699). The last anti-CD123 antibody discussed in this review is the ADC IMGN632 which comprises a humanized anti-CD123 antibody linked to a DNA-alkylating agent. Pre-clinical studies showed its efficacy against AML cell lines, against primary human AML samples and in xenograft models. Notably, the compound demonstrated potent activity against AML samples at concentrations which were significantly lower than concentrations affecting healthy hematopoietic progenitor cells [168]. IMGN632 is currently evaluated in a clinical trial in patients with relapsed or refractory AML, BPDCN, MPN and ALL (ClinicalTrials.gov Identifier: NCT03386513).

Since clinical trial results with anti-CD123 antibodies do differ significantly, it remains to be investigated which anti-CD123 antibody and which formulation has the potential to be used in clinical applications or if other strategies to target CD123 might be superior.

## 8. Tumor Vaccination

The potency of typical MPN mutations, including *JAK2^V617F^* or *CALR* exon 9 mutations, for cancer vaccination therapy as a specific anti-cancer immunotherapy approach was pushed forward by findings of Holmström et al. who could identify a spontaneous T-cell response against a PD-L1-derived epitope in MPN patients [169,170,171]. Patients with mutated spliceosome regulators showed disease-specific splicing abnormalities and typical driver mutations in the *CALR*, *JAK2* and *MPL* genes were hypothesized to be a rich and promising source for neoantigens [172]. Based on their findings, both *JAK2^V617F^* and *CALR* exon 9 mutation epitopes are recognized by T-cells, making these mutations promising targets for cancer immune therapy and anti-tumor vaccination [173]. They did claim that 71% of all analyzed MPN patients did display a strong immune response against PD-L1, whereas the PD-L1 specific T-cell response was stronger in patients with non-advanced MPN compared to patients with advanced MPN [169]. Following these findings, they initiated a phase I first-in-human study to monitor the safety, toxicity and Immunological response to vaccination in patients with *CALR*-mutant MPN vaccinated with a CALR exon 9 mutated peptide (ClinicalTrials.gov Identifier: NCT03566446). The outcome will be measured by AE grading and T-cell cytokine release towards the target antigens. So far, no results were published. The selected trials are summarized in Table 3. The idea of a vaccination therapy and strong immune response against mutant CALR is supported by recent findings about the potency of secreted mutant CALR acting as a cytokine to interact and bind to the thrombopoietin receptor on neighboring MPN-cells. It can thereby specifically stimulate JAK2/STAT5 signaling in these mutant cells. Pecquet et al. found that mutant CALR is secreted in MPN patients in high levels and that it can form complexes with the thrombopoietin receptor. Binding to the thrombopoietin receptor and subsequent activation of JAK/STAT signaling could stimulate the expansion of the mutant MPN clone [174]. Besides the finding about specific T-cell responses against these major MPN mutations, there was also one study proposing an activation of Arginase-1-specific T-cells could be a novel immunotherapeutic strategy to target immunosuppressive and malignant MPN-cells and to combine this strategy with further immunotherapeutic compounds [175]. Indeed, Arginase-1 does not only play a pivotal role in metabolic pathways but also regulates the immune response by depleting arginine in the microenvironment and thereby limiting the activation of T-cells and suppressing the immune response [175,176,177]. In line with its physiological immunosuppressive role, it could be shown that MPN patients have increased levels of Arginase-1 expressing MDSCs, thereby pathophysiologically suppressing the anti-leukemia immune response in these patients [98]. To the best of our knowledge, all the studies about reactive T-cells against mutant proteins frequently found in MPN patients were only done in vitro, so it is unclear how relevant these results are in vivo. Besides JAK2 and CALR-mutant epitopes, a recent study did elucidate the mutational landscape of Ph-negative MPNs with regard of putative novel targets for immunotherapy. Based on gene fusions, single nucleotide variants and insertions and deletions, the group generated a virtual peptide library of 149 unique neoantigens in 62% of all analyzed MPN patients. They proposed that splicing defects due to mutations of the splicing factor *SF3B1* could even offer a broader repertoire of yet unknown neoantigens in MPN. The findings could be used as basis for the development of personalized anti-tumor vaccine therapies and a vaccination-based therapeutic intervention could therefore represent a potential new therapy to enhance the body’s own specific anti-tumor immune response [172]. Since PD-1 blockade was shown to expand mutation-associated, antigen-specific tumor-reactive T-cell clones in the peripheral blood in lung cancer patients, it was hypothesized that immune checkpoint blockade could be beneficial to combine with anti-tumor vaccines to enhance the anti-leukemia immune response [6,178].

In addition to vaccination therapy in MPN, it was recently discussed that cancer vaccination might be synergistically combined with HMAs to overcome the unmet need for a curative treatment in MDS patients. Although allo-HSCT is the only curative treatment option to date, the high treatment-related mortality often makes it not feasible [6]. Cancer testis antigens (CTAs) encode immunogenic proteins which are usually expressed by germ cells only, but not by healthy adult cells. It was found that tumor cells do also express these genes [179,180]. Application of HMAs, such as Azacitidine or Decitabine, did cause enhanced CTA expression on tumor and leukemia cells without affecting their expression on normal healthy cells [180,181,182,183,184]. It was proven previously in a patient study that treatment with HMAs increased the expression of CTAs, thereby stimulating a specific cytotoxic T-cell response [185]. Since CTAs are not found in healthy adult tissue and are highly immunogenic, they were hypothesized to be a new class of target molecules in cancer immunotherapeutic approaches and anti-tumor vaccination therapy, whereas the therapeutic response could even be enhanced upon combination with HMAs [6]. Besides an HMA-mediated increased inflammatory response in malignant cells, these compounds were also shown to deplete MDSCs which are found in high numbers in MDS patient bone marrow [112]. Clinical trials testing the potency of vaccination targeting CTAs either alone or in combination with HMAs are emerging, whereas most of them were designed to target solid tumors and not myeloproliferative diseases. Although the treatment regimen was well tolerated, the clinical response was only limited [186]. A phase 2 study did use a WT1 peptide vaccine for the treatment of AML and ALL patients and could highlight that this treatment increases a specific immune response and can increase the survival of patients (ClinicalTrials.gov Identifier: NCT01266083) [187]. With the aim to improve successful therapies for MDS patients, a phase I trial is currently evaluating the efficacy of a combination of HMAs and experimental peptide vaccination against the NY-ESO-1, PRAME, MAGE-A3, and WT-1 tumor antigens in patients with high-risk MDS and AML (ClinicalTrials.gov Identifier: NCT02750995). Since HMA treatment did increase the expression of immune checkpoint molecules PD-L1, PD-L2, PD-1, and CTLA4 in MDS and AML patients, a triple therapy combining tumor-specific vaccination peptides, HMAs and immune checkpoint blockade is hypothesized to significantly enhance the anti-leukemia immune reaction [188,189]. The concept is currently evaluated in a clinical trial (ClinicalTrials.gov Identifier: NCT03358719). To the best of our knowledge, there were no results published yet for the two last described trials.

## 9. Immune Checkpoint Blockade

Bozkus et al. described a novel concept of blocking immune checkpoints in patients with *CALR*-mutant MPN to increase the T-cell immune response against the myeloid disorder [190]. They could identify a specific T-cell reactivity against neoantigens in *CALR*-mutant MPN in some patients [190]. Additionally, naïve T-cells isolated from healthy donor peripheral blood mononuclear cells (PBMCs) did show effector functions after priming with mutant CALR peptides, whereas they did not exhibit any cytotoxic function against the corresponding WT peptide. Incubation of T-cells with the mutant peptide increased T-cell proliferation and production of IFNγ and TNFα in the effector cells [191]. The up-regulation of programmed cell death protein 1 (PD-1) and cytotoxic T lymphocyte antigen 4 (CTLA-4) on the cell surface did attenuate the specific immune response in other patients. After blockade of both immune checkpoint molecules ex vivo and an increased T-cell reactivity, they also applied immune checkpoint blockade (ICB) in patients with monoclonal antibodies which restored the specific T-cell response at least in some *CALR*-mutant MPN patients. Since the CALR neoantigen stimulates responses from CD4 and CD8 T-cells, they further hypothesized that this molecule could serve as a vaccine to improve MPN therapy [190]. A major concern for successful anti-PD-1 immunotherapy is T-cell exhaustion. The analysis of MPN patients revealed that 71% of MPN patients display a significant immune response against the programmed death-ligand 1 (PD-L1), whereas patients with advanced MPN have significantly fewer and weaker PD-L1 specific immune responses compared to patients with non-advanced MPN [169]. In naïve mice without exhausted T-cells, ICB reduced MPN disease burden and improved survival [192].

For many years, the molecular mechanism by which mutant HSPCs, as well as pre-leukemic cells can escape from the control through the body’s immune system remained unclear. In 2018, Prestipino et al. described that oncogenic *JAK2^V617F^* mutations cause the up-regulation of PD-L1 in myeloproliferative neoplasms, thereby enabling these cells to escape from the immune system [192]. They could show that mutant *JAK2* caused the phosphorylation of STAT3 and STAT5, thereby enhancing *PD-L1* promoter activity causing increased PD-L1 protein levels on *JAK2^V617F^* mutant myeloid cells. Up-regulated PD-L1 in turn attenuated cytotoxic T-cell activity and affected T-cell metabolism and cell-cycle activity which could be reversed by JAK2 inhibition or PD-1 blockade [192]. In general, PD-L1 binds to its receptor PD-1 which is located on T-cells, thereby stimulating T-cell attenuation, reduced cell-cycle progression and T-cell exhaustion [193,194]. It was reported before that tumor cells engage the PD-1 ligand on their surface to evade from the control through the body’s immune system [195]. Consistent with the high PD-L1 expression observed, *JAK2^V617F^*-MPN was susceptible to PD-1 blockade, which was dependent upon T-cells, in human MPN xenografts, in a *JAK2^V617F^*-driven mouse model and in one MPN patient who relapsed after allo-HSCT [196]. The findings described by Prestipino et al. summarize a novel immunotherapeutic concept for MPNs based on the oncogene-driven immune escape of *JAK2^V617F^*-mutant cells via the JAK/STAT/PD-L1 axis [192].

A phase II trial on Nivolumab for patients with PMF, post-ET MF, or post-PV MF has been performed (ClinicalTrials.gov Identifier: NCT02421354). Although this clinical trial on the efficacy of PD-1 blockade using the monoclonal antibody Nivolumab was prematurely terminated due to a lack of efficacy, more clinical studies are currently ongoing to evaluate if ICB could be a promising therapeutic option in MPN patients (Table 4). Based on pre-clinical findings, the role of PD-L1 blockade in patients with primary MF, post-PV MF or post-ET MF was planned to be evaluated using the PD-L1 antibody Durvalumab (ClinicalTrials.gov Identifier: NCT02871323). However, the study was withdrawn before any patients were enrolled. One ongoing phase II study is testing the effectiveness of PD-1 inhibition with the anti-PD1 antibody Pembrolizumab in advanced MPN, chronic phase (MF-CP), accelerated phase (MPN-AP), or blast phase (MPN-BP) (ClinicalTrials.gov Identifier: NCT03065400). Pembrolizumab is FDA approved for metastatic melanoma and is tested at a dose of 200 mg administered via intravenous infusion over 30 min, given every 3 weeks. One treatment cycle is 3 weeks and the study is planned for a time of 6 treatment cycles. Additional to the clinical outcome, exploratory biomarkers will be taken from patients at baseline, cycle 3, and cycle 7 and at 1 year of therapy. If patients show a clinical improvement after 6 cycles of therapy, they will continuously receive Pembrolizumab until evidence of disease progression, unacceptable toxicity, and patient or physician decision for a maximum of 2 years. To date of this review writing, no results were published in this study. Besides targeting the PD-1/PD-L1 axis in MPN, another clinical trial (phase I/Ib) aims to study the side effects, toxicity and best dose of the CTL-A4 inhibitor Ipilimumab or the PD-1 inhibitor Nivolumab in patients with hematologic malignancies. Different to other known studies, this trial only recruits patients who relapsed after allo-HSCT (ClinicalTrials.gov Identifier: NCT01822509). The investigators claim that immunotherapy with monoclonal blocking antibodies could help the patient’s own immune system to control cancer growth. To the best of our knowledge, there were no results published yet in this clinical trial. One phase I clinical trial was conducted to evaluate if CTLA-4 blockade using Ipilimumab is beneficial after HSCT for the treatment of patients with persistent, relapsed or progressive cancer (ClinicalTrials.gov Identifier: NCT00060372). Ipilimumab was found safe to use but might induce organ-specific immune-related adverse events (irAEs) at higher doses. The treatment did neither cause graft rejection, nor increased GvHD severity. However, the treatment efficacy was limited and further evaluation is necessary to find a suitable treatment schedule with maximal clinical efficacy and low irAEs [197]. To further characterize the efficacy of PD-1 blockade in relapsed/refractory AML, a phase II trial was conducted on the efficacy of standard high dose chemotherapy Cytarabine followed by Pembrolizumab (ClinicalTrials.gov Identifier: NCT02768792). The overall complete response rate was 35%, whereas 56% of these patients did not have any evidence of MRD. Grade II aGvHD and moderate cGvHD was seen in 50% of the enrolled and treated patients. Patients in CR did show a significantly increased T-cell receptor diversity in the peripheral blood. It could be concluded that high dose chemotherapy followed by PD-1 blockade is well tolerated and shows encouraging response rates in high-risk patients without any additive toxicity after HSCT. Moreover, the treatment did increase the B- and T-cell diversity in the peripheral blood. Three additional studies are ongoing to evaluate if the anti-PD-1 antibody Nivolumab has a beneficial effect for the treatment of AML, either as monotherapy or in combination with chemotherapy or HMAs. A phase II trial is studying the application of Nivolumab in AML patients currently being in remission but with a high risk of relapse (ClinicalTrials.gov Identifier: NCT02532231). The investigators hypothesize that ICB could help the body’s immune system to attack remaining tumor cells thereby preventing tumor growth and spread. The first update was published in 2018 and the authors concluded that Nivolumab is well tolerated, safe and feasible in high-risk AML to prevent relapse [198]. The second clinical trial (phase II) is evaluating the side effects and best dose of Nivolumab and Azacitidine with or without the anti-CTLA-4 antibody Ipilimumab in patients with treatment-refractory, relapsed or newly diagnosed AML (ClinicalTrials.gov Identifier: NCT02397720). The combination of Nivolumab and Azacitidine showed an ORR of 33%, whereas 22% of all enrolled patients were in CR. The addition of Ipilimumab had an encouraging CR (43%) but more patients need to be included into the trial. Nevertheless, a combination therapy could be superior over ICB monotherapy [199]. The third clinical trial which is testing ICB in combination with other compounds is combining the chemotherapeutics Idarubicin and Cytarabine with Nivolumab in patients with high-risk MDS and AML (ClinicalTrials.gov Identifier: NCT02464657). The results were published in 2019 and it was found that a combination therapy is feasible in patients with high-risk MDS or newly diagnosed AML and about 43% of all analyzed patients achieved a response and could proceed to allo-HSCT. However, the authors hypothesized that an earlier initiation of ICB could further improve the beneficial effects [200].

## 10. WT1-Specific T-Cells

The classical forms of MPN are mostly characterized by mutations of *JAK2*, *CALR*, and MPL, as described previously [201]. Moreover, Cottin et al. found that Wilms’ Tumor Antigen 1 (WT1) is overexpressed in MPN patients compared to healthy subjects [201]. A gene expression analysis of 152 patients at time of diagnosis revealed that *WT1* expression was significantly higher in PMF patients compared to ET and PV patients or healthy controls. Moreover, the expression increased during myelofibrotic transformation and high *WT1* transcript levels could be linked to splenomegaly and thrombocytopenia and overexpression of *WT1* was hypothesized to play an important role in the leukemic transformation of MPN [201,202]. Comparable with the results reported about *WT1* expression in MPN, its expression is also higher in leukemia cells and LSCs compared to normal healthy hematopoietic cells and is a possible prognostic factor to predict clinical outcome and to detect MRD [203,204,205,206]. WT1 is a possible antigen to specifically target in leukemia patients as it was demonstrated that WT1-reactive cytotoxic T-cells mediate a strong anti-tumor immune response in post-transplant patients [207]. The expansion of these cells was correlated with an increased GvL reaction in patients with ALL [208]. Further strategies, including the transfer of WT1-specific T-cells or autologous vaccination of AML patients with the WT1 peptide, did increase anti-leukemia immune responses in relapsed or high-risk leukemia patients [203,207,209]. These findings drew attention to the transfer of WT1-specific cytotoxic T-cells into AML patients. Chapuis et al. isolated a high-affinity WT1-specific T-cell receptor (TCR) from normal donor repertoires and inserted it into donor cytotoxic T-cells [206]. The engineered T-cells were prophylactically transferred into AML patients after HSCT and 100% relapse-free survival was observed at a median of 44 months following T-cell transfusion. The comparison group had only 54% relapse-free survival (ClinicalTrials.gov Identifier: NCT01640301) [206]. In a second study, Kim et al. demonstrated that in vitro generation of WT1-specific cytotoxic T lymphocytes and subsequent transfer is a feasible therapeutic approach for the treatment of AML patients being at high risk of relapse after allo-HSCT [203]. Summarizing these studies, the transfer of cytotoxic T-cells specifically targeting the WT1 antigen together with allogeneic or autologous T-cells in high-risk AML patients could be a promising strategy to enhance a strong and specific anti-leukemia immune reaction and to prevent AML recurrence in these patients [203,206].

## 11. Conclusions

During the last years, an impressive progress has been made in understanding the molecular mechanisms driving MPN development and leukemic transformation. Moreover, the role of pro-inflammatory cytokines and immunosuppressive myeloid cells in the bone marrow of MPN patients in mediating leukemia immune escape was elucidated in detail. Multiple studies are ongoing to evaluate novel treatment strategies aiming to overcome the immunosuppressive mechanisms and to enhance an anti-leukemia immune response. Although allo-HSCT is still the only potentially curative treatment for most patients suffering from myeloid malignancies, there are promising novel strategies effectively incorporating immunotherapeutic strategies to overcome the unmet need for an effective treatment for patients with hematologic malignancies. However, myeloid tumors were found to exhibit a variety of strategies to successfully undergo immune evasion making effective immunotherapy difficult. Nevertheless, target-specific cytotoxic T-cell transfer, hypomethylating agents, ICB, specific antibodies and tumor vaccination are promising novel immunotherapeutic approaches against myeloid disorders. The successful treatment will always depend on the detailed understanding of the underlying disease, its microenvironment, genetic aberrations, and potential immune escape mechanisms to successfully treat patients either as monotherapy or in a combination approach.

## Figures and Tables

**Table 1:** Selected Clinical Trials of IFN-α2a therapy in MPN.

Trial	Treatment	Diagnosis	Outcome Measures	Status, Response, Comments
**NCT02910258**	Pegylated interferon-α2a	PMF, SMF	Primary: ORR; Secondary: OS, AE	Completed; observational study; IFNα2a might improve OS, and leukemia free survival; IFNα2a increased risk of GvHD if given before allo-HSCT
**NCT00241241** **(Phase II)**	Pegylated interferon-α2a	PV; previously untreated patients or treated with phlebotomy or HU	Primary: ORR; Secondary: safety, molecular response	Completed; Decrease of JAK2 allele frequency in 89% of patients; peg-IFN-α2a targets mutant clone; molecular CR in 7 patients; low toxicity
**NCT02742324** **(Phase I/II)**	Ruxolitinib; Pegylated interferon-α2a	PMF, SMF	Primary: safety, DLT; Secondary: Molecular response	Recruiting; No DLT, well-tolerated combination therapy; decreased spleen size and JAK2 allele burden; improvement in blood counts; 63% with complete hematological response; decrease of other driver mutations
**NCT01259856** **(Phase III)**	PEGASYS (peg-IFN-α2a); Hydroxyurea; Aspirin	High-risk PV and ET	Primary: CR, PR; Secondary: AE, change in TSS, JAK2 allele burden, disease progression, death	Completed; ORR at 12 months was 69.2% (ET) and 60% (PV); CR was higher in CALR-mutant ET compared to CALR non-mutant; significant rate of AE (manageable); PEG for patients being intolerant or resistant to HU

AE: adverse events; allo-HSCT: allogeneic hematopoietic stem-cell transplantation; CR: complete remission; DLT: dose-limiting toxicity; ET: essential thrombocythemia; GvHD: Graft-versus-Host Disease; ORR: overall response rate; OS: overall survival; PMF: primary myelofibrosis; PR: partial remission; PV: Polycythemia vera; SMF: secondary myelofibrosis.

**Table 2:** Selected Clinical Trials of CD123-directed therapy in myeloid malignancies.

Trial	Treatment	Diagnosis	Outcome Measures	Status, Response, Comments
**NCT02268253** **(Phase II)**	Tagraxofusp (SL-401; CD123-directed cytotoxin)	R/R MF, advanced MF, high-risk MF, CMML	Primary: AE, ORR	Recruiting; Tagraxofusp has single-agent activity; well tolerated
**NCT04317781** **(Phase II)**	Tagraxofusp (CD123-directed cytotoxin)	BPDCN after autologous or allogeneic HSCT	Primary: AE; Secondary: PFS, OS	Recruiting
**NCT04342962** **(Phase II)**	Tagraxofusp (CD123-directed cytotoxin)	R/R CD123^+^ AML, BPDCN-IF	Primary: ORR; Secondary: AE, OS, EFS,	Not yet recruiting (estimated July 2020)
**NCT01632852** **(Phase I)**	CSL362 (Anti-IL3Rα/Anti-CD123 Monoclonal Antibody)	CD123^+^ AML in CR or CR with incomplete platelet recovery at high risk of early relapse	Primary: AE, DLT; Secondary: PK, immunogenicity	Completed; Study to generate dose and dosing schedule
**NCT02472145** **(Phase II/III)**	Decitabine (HMA); Talacotuzumab (anti-CD123)	R/R AML, de novo AML, patients not eligible for curative therapy	Primary: CRR, OS; Secondary: EFS, ORR, DOR, CR	Completed; Lack of efficacy and high toxicity of combination therapy
**NCT02848248** **(Phase I)**	SGN-CD123A (anti-CD123 ADC)	R/R AML	Primary: AE, DLT, LA; Secondary: PK, immunogenicity, OS, ORR	Terminated
**NCT02181699** **(Phase I)**	KHK2823 (anti-CD123)	R/R AML, R/R MDS, patients not eligible for curative therapy	Primary: AE; Secondary: PK, ORR, OS, EFS, RFS, PFS, DFS, immunogenicity, PD	Terminated; Failed treatment response
**NCT03386513** **(Phase I/II)**	IMGN632 (anti-CD123, DGN549 ADC)	R/R AML, R/R BPDCN, R/R ALL, high-risk MDS, MPN, CMML	Primary: MTD, RP2D; Secondary: AE, ORR, PK, Immunogenicity	Recruiting; Objective responses in one third of patients during initial dose escalation; no DLT

AE: adverse events; ADC: antibody–drug conjugate; AML: Acute myeloid leukemia; ALL: Acute lymphoblastic leukemia; BM: bone marrow; BPDCN: blastic plasmacytoid dendritic cell neoplasm; CML: chronic myeloid leukemia; CMML: chronic myelomonocytic leukemia; CRR: complete response rate; DFS: disease-free survival; DLT: dose-limiting toxicity; EFS: event-free survival; MF: Myelofibrosis; MPN: myeloproliferative neoplasm; MTD: maximum tolerated dose; ORR: overall response rate; OS: overall survival; PB: peripheral blood; PFS: progression-free survival; PK: pharmacokinetics; PR: partial remission; RP2D: recommended phase 2 dose; R/R: relapsed/refractory.

**Table 3:** Selected Clinical Trials investigating tumor vaccination in myeloid malignancies.

Trial	Treatment	Diagnosis	Outcome Measures	Status, Response, Comments
**NCT03566446** **(Phase I)**	CALRLong36 peptide (Ex 9 mut) vaccine	CALR-mutant MPN (ET, PMF, MPN unclassifiable)	Primary: AE; Secondary: Immune response (T-cell cytokine release), mutation status, ORR	Active; No trial results posted yet; CALRLong36 peptide did show prompt responses in vitro
**NCT01266083** **(Phase II)**	WT1 peptide vaccine	AML, ALL; patients being in CR; patients with WT1+ disease	Primary: AE, OS; Secondary: DFS, Immunol. ogic response, effects on MRD, OS	Completed; Vaccine was well tolerated; AEs: injection site reaction, fatigue, skin induration; vaccine-stimulated specific immune response
**NCT02750995** **(Phase I)**	NPMW-peptide vaccine (against long peptide sequences from NY-ESO-1, PRAME, MAGE-A3, WT-1); Azacitidine	High-risk MDS, AML (<30% blasts)	Primary: AE; Secondary: specific T-cell reactivity, ORR	Recruiting
**NCT03358719** **(Phase I)**	DEC-205/NY-ESO-1 Fusion Protein CDX-1401; Decitabine; Nivolumab	AML (<30% blasts), MDS, high-risk MDS, CMML, refractory anemia	Primary: AE; Secondary: immune profile, PB and BM response, CRR, PRR	Active; final data collection for primary outcome measure

AE: adverse events; AML: Acute myeloid leukemia; ALL: Acute lymphoblastic leukemia; BM: bone marrow; CMML: chronic myelomonocytic leukemia; CR: complete remission; CRR: complete response rate; DFS: disease-free survival; MDS: Myelodysplastic Syndrome; MPN: myeloproliferative neoplasm; ORR: overall response rate; OS: overall survival; PB: peripheral blood; PMF: primary myelofibrosis; PRR: partial remission rate.

**Table 4:** Selected Clinical Trials on ICB in myeloid malignancies.

Trial	Treatment	Diagnosis	Outcome Measures	Status, Response, Comments
**NCT02421354** **(Phase II)**	Nivolumab (anti-PD-1)	Hepatomegaly, MF transformation in ET, PV, PMF, Splenomegaly	Primary: efficacy in MF; Secondary: AE; Tertiary: time to response, symptom burden, BM fibrosis, JAK2 allele burden	Terminated; 8 patients enrolled; terminated due to serious AE (75%) and other AE (87.5%)
**NCT02871323** **(Phase I)**	Durvalumab (anti-PD-L1)	PMF, PV	Primary: AE; Secondary: MF symptom burden, response in PB and BM, cytokine profile	Withdrawn before enrollment of patients
**NCT03065400** **(Phase II)**	Pembrolizumab (anti-PD-1)	Chronic phase MF, PMF, post-ET MF, PV, MPN-AP/BP	Primary: clinical improvement; Secondary: MPN-AP/BP patients that achieve complete morphologic remission of blasts	Completed; No results posted yet
**NCT01822509** **(Phase I/Ib)**	Ipilimumab (anti-CTLA-4), Nivolumab (anti-PD-1)	Patients relapsed from hematologic malignancies after HSCT (MPN, ALL, AML, CLL, CML, MDS, Hodgkin lymphoma, Non-Hodgkin lymphoma)	Primary: MTD, DLT, AE; Secondary: clinical response, PFS, OS, immune cell numbers, cytokine production	Active; 21% AE; 14% Ipilimumab discontinuation (GvHD); 23% CR, 9% PR, 27% decreased tumor burden; infiltration of CD8 T-cells, decreased T_reg_ activation; Nivolumab: 23% PFS, 56% OS; severe AE and GvHD
**NCT00060372** **(Phase I)**	Ipilimumab (anti-CTLA-4); Donor lymphocytes	Patients with persistent or progressive cancer after allogeneic stem-cell transplant	Primary: incidence of aGvHD, graft rejection, immune reaction; Secondary: cGvHD, DFS, OS, ORR, T-cell activation	Completed; Induction of graft-versus-tumor effects after HSCT; Ipilimumab safe to use and causes anti-tumor response
**NCT02768792** **(Phase II)**	Cytarabine (HiDAC); Pembrolizumab (anti-PD-1)	R/R AML	Primary: CR; Secondary: AE, PR, CR, RFS, PFS, OS	Active; 46% ORR, 38% CR/CRi, OS 8.9 months, DFS 5.7 months
**NCT02532231** **(Phase II)**	Nivolumab (anti-PD-1)	AML in remission at high risk of relapse	Primary: recurrence-free survival; Secondary: Immunol. ogic response, OS, AE	Recruiting; OS 86% (12 months) and 67% (18 months); therapy well tolerated; detectable MRD while on therapy
**NCT02397720** **(Phase II)**	Azacitidine, Ipilimumab (anti-CTLA-4), Nivolumab (anti-PD-1)	R/R AML, newly diagnosed AML	Primary: MTD, ORR, AE; Secondary: DFS, OS, PFS	Recruiting; 21% CR/CRi, 26% BM blast reduction (>50%), 23% disease progression; 12–14% AE; pts with CR: higher CD8 Tc infiltration into BM; well tolerated
**NCT02464657** **(Phase I/II)**	Idarubicin, Cytarabine, Nivolumab (anti-PD-1), Solu-medrol, Dexamethasone	High-risk MDS, AML	Primary: MTD; Secondary: EFS	Active; Addition of Nivolumab to chemotherapy feasible in AML or high-risk MDS patients; GvHD needs to be improved

AE: adverse events; aGvHD: acute Graft-versus-Host Disease; allo-HSCT: allogeneic hematopoietic stem-cell transplantation; AML: Acute myeloid leukemia; ALL: Acute lymphoblastic leukemia; BM: bone marrow; cGvHD: chronic Graft-versus-Host Disease; CLL: Chronic lymphatic leukemia; CML: chronic myeloid leukemia; CMML: chronic myelomonocytic leukemia; CR: complete remission; DLT: dose-limiting toxicity; EFS: event-free survival; ET: Essential thrombocythemia; MDS: Myelodysplastic Syndrome; MPN: myeloproliferative neoplasm; MPN-AP/BP: accelerated/blast phase MPN; MRD: minimal residual disease; MTD: maximum tolerated dose; ORR: overall response rate; OS: overall survival; PFS: progression-free survival; PMF: primary myelofibrosis; R/R: relapsed/refractory.
